# Optimization reconstruction method of object profile using flexible laser plane and bi-planar references

**DOI:** 10.1038/s41598-018-19928-4

**Published:** 2018-01-24

**Authors:** Guan Xu, Jing Yuan, Xiaotao Li, Jian Su

**Affiliations:** 1 Traffic and Transportation College, Nanling Campus, Jilin University, Renmin Str. 5988#, Changchun, China; 2 School of Mechanical Science and Engineering, Nanling Campus, Jilin University, Renmin Str. 5988#, Changchun, China

## Abstract

An optimization method to reconstruct the object profile is performed by using a flexible laser plane and bi-planar references. The bi-planar references are considered as flexible benchmarks to realize the transforms among two world coordinate systems on the bi-planar references, the camera coordinate system and the image coordinate system. The laser plane is confirmed by the intersection points between the bi-planar references and laser plane. The 3D camera coordinates of the intersection points between the laser plane and a measured object are initially reconstructed by the image coordinates of the intersection points, the intrinsic parameter matrix and the laser plane. Meanwhile, an optimization function is designed by the parameterized differences of the reconstruction distances with the help of a target with eight markers, and the parameterized reprojection errors of feature points on the bi-planar references. The reconstruction method with the bi-planar references is evaluated by the difference comparisons between true distances and standard distances. The mean of the reconstruction errors of the initial method is 1.01 mm. Moreover, the mean of the reconstruction errors of the optimization method is 0.93 mm. Therefore, the optimization method with the bi-planar references has great application prospects in the profile reconstruction.

## Introduction

The vision measurement including structured light is an effective non-contact 3D measurement method^1–7^. As a laser projector provides more strong and narrowband illumination than a normal digital projector, the laser projector is considered as a flexible symbol for projecting a laser plane onto the object to be measured^8,9^. The laser curve between the measured object and the laser plane contains the depth information about both the intersection position and the measured object. Therefore, the measurement system based on the structured light is favored by many researchers.

The vision measurement based on the structured light is widely studied due to the advantages of wide measurement range, reasonable test speed and precision in visual measurement approaches^10–12^. Huynh^13^ presents a calibration method for the structured light system derived from the projector. Three collinear world points contribute the cross ratio value in the image, which is the same with the cross ratio calculated by the world points. The recovery matrix of the stripe plane is provided according to the projection from the structured light to the image. A double cross ratio method is also reported by Wei^14^ to achieve the calibration of the structured-light-stripe vision sensor. Wei^15^ employs the vanishing points and vanishing lines, which are derived from a target with parallel lines, to improve the laser plane calibration. Li^16^ designs a flexible laser scanning system including an industry robot arm and a laser scanner. The rotations and translations of the robot arm are considered in the scanning model. Niola^17^ describes a calibration for the laser scanner in robotic applications. A target with the known movement is employed to model the geometry of the laser emitter and camera. An approach is illustrated by Le^18^ by fusing the structured illumination with the data to reconstruct the 3D sharp edge and realize the automatic inspection and reconstruction. In addition, an algorithm is introduced to reconstruct the 3D object profile with the sharp edge. A reconstruction method of 3D profile is proposed by Ma^19^ on the basis of a nonlinear iterative optimization to reduce the errors from the lens distortion. According to the shape of projection light, the vision measurement methods based on the structured light can be divided into four parts: point structured light, line structured light, grating structured light and coded structured light. The point structured light method can obtain the 3D data of a point. However, it is slow for the large object measurement. The methods of grating or coded structured light are generally based on the digital light processing (DLP) projector and the camera^20,21^. Villa^22^ proposes a reference-plane based method to reconstruct the 3D data on the object. The depth is performed by moving the flat plate on a linear stage. Then, a pattern of crossed gratings along the *x* and *y* directions is projected on the measured object. The *x* and *y* coordinates are determined by the fringe phases generated from the Fourier transform and the inverse Fourier transform. Zhang^23^ presents a method considering the digital projector as a camera. The digital micro-mirror device (DMD) image is established by the mapping between the (Charge Coupled Device) CCD pixels and DMD pixels. Then the intrinsic parameter matrix of the digital projector is calibrated as the matrix of a camera. The extrinsic parameter matrices are derived from the Zhang’s method. Finally, the 3D coordinates are reconstructed by the image coordinates and the extrinsic parameter matrices. Hu^24^ introduces an approach to calibrate the projector-camera system. The absolute phase map is solved by a three-step algorithm. Then, a flat plate is designed on a linear stage and driven by a stepper motor. By moving the flat plate and the projection images, four unknown parameters are calibrated for the measurement system. Finally, the system parameters are optimized by a coordinate measurement machine (CMM), a plate with holes and an iterative algorithm. The digital-projector-based methods take the advantages of high efficiency and plenty of data. However, as the pattern of the digital projector is generated from a light bulb, the coded light pattern of the digital projector tends to be impacted by the illuminations from environment, such as the sunlight. After the calibration, the relative position between the digital projector and the camera is fixed. As the planar laser projector contributes the narrow-band and high-density structured light, it is appropriate to measure the complex surface with a moderate test speed, by avoiding the environmental interference illuminations.

Camera calibration is the basis of the accuracy of a vision measurement system. 3D recovery depends on the single or multi-view images captured by a camera. Therefore, the camera calibration has also attracted researchers to improve the 3D reconstruction accuracy. At present, the calibration methods are mainly divided into 1D calibration^25,26^, 2D calibration^27–29^ and 3D calibration^30^. The 1D calibration reference is simple in structure and easy to be manufactured, but the calibration accuracy is the lowest due to the lack of information on the 1D reference. Although the structure of the 2D calibration reference is a little more complicated than the one of the 1D reference, it is convenient to be made and moved to different places with the advantage of high calibration accuracy. The 3D calibration reference provides the highest accuracy for the camera calibration. Nevertheless, the 3D calibration reference is complex to be made and suitable for specific situations. Although a 2D reference can be used to calibrate the projection laser plane^31^, many images of the planar reference in different positions should be captured to calibrate only one laser plane. One target can provide sufficient number of features for solving the intersection points, when the relative position between the camera and the projector is fixed in previous work. We focus on the other situation, which is to reconstruct the intersection points from a flexible laser plane. There is only one intersection line between the flexible laser plane and the target. As the flexible laser plane is determined by at least two lines on the plane, a reconstruction method adopting the bi-planar references is proposed to perform the laser plane calibration in the camera coordinate system. The bi-planar method with two planar references contributes four main advantages that belong to the 2D reference and 3D reference. As the two planar references locate on different planes, the feature points on the bi-planar references are similar to the ones on the 3D reference. Thus, it should be noted that the calibration and reconstruction are achieved by only one image of the camera. Moreover, the calibration reference consists of two planar references, which are easier to be manufactured than the 3D references. Then, the large 3D or 2D references are complicated to be prepared for the profile measurement in a large view field. The reconstruction method of the bi-planar references is easy to be extended to the method of *n*-planar references. The *n*-planar references method extends the effective measurement in a larger view field. However, the large 3D or 2D references for a larger view field are replaced by the small 2D references that are convenient to be realized in the reconstruction. Finally, the laser plane is flexible, e.g. hand-held, as the laser plane that is projected to the bi-planar references can be solved in only one image.

An optimization reconstruction method of the object profile is proposed for the vision measurement using the flexible planar laser and bi-planar references. Two planar references are separately distributed at the left and right sides of the measured object. The two planar references are non-coplanar in space. First, the laser plane, generated from a laser projector, is projected onto the measured object. Therefore, the laser plane intersects with the object as well as the planar references providing an intersection curve and two intersection lines. Then, in the camera coordinate system, the flexible laser plane is modeled by the projections of feature points on the two planar references and the image coordinates of the laser intersection points. Finally, the 3D coordinates of the measured object are determined by the projection of the intersection curve and further enhanced by the optimization function. The optimization method is compared with the initial method by the reconstructed distance errors to verify the accuracy of the reconstruction method.

## Methods

**Figure 1:**
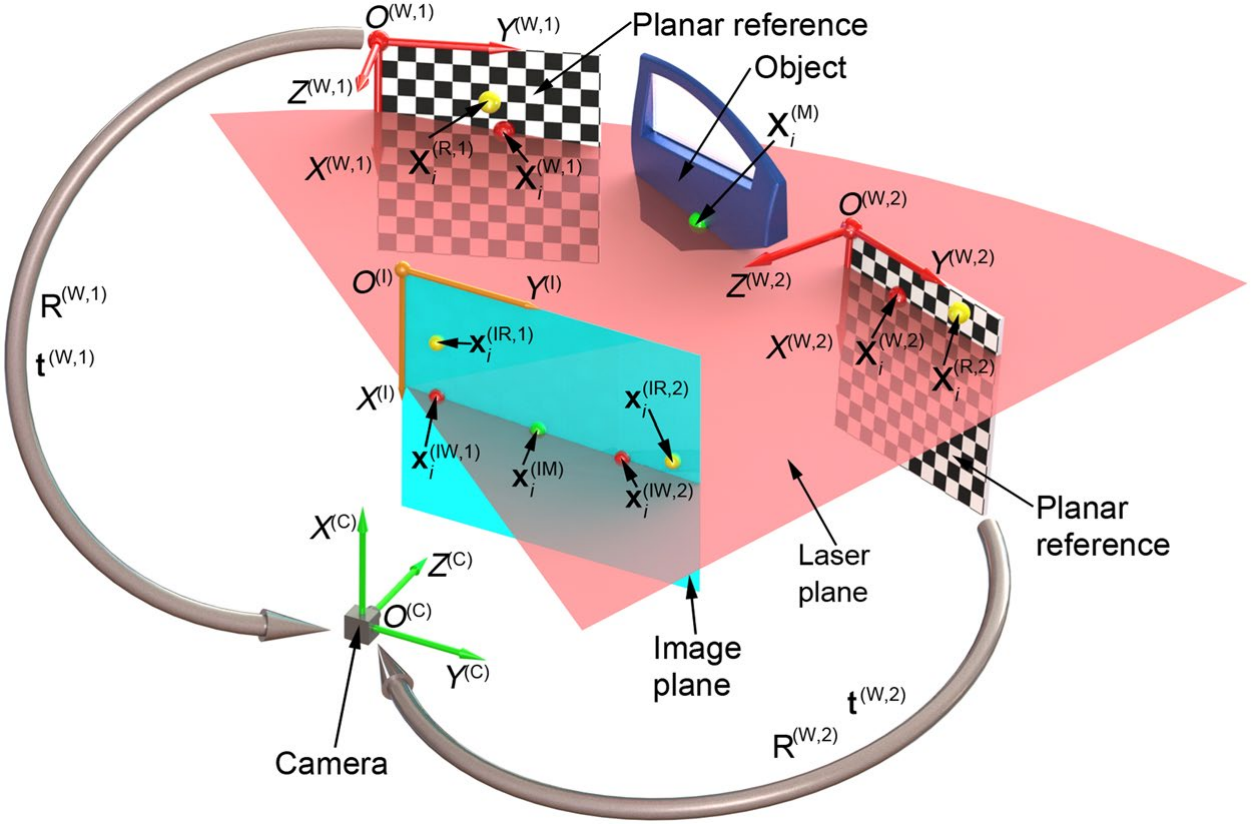
The reconstruction principle with a flexible laser plane and two planar references in the view field of a camera.

For the 3D profile reconstruction problem, a random laser plane is projected to the measured object and two planar references in Fig. 1. Two world coordinate systems *O*^W1^-*X*^W1^*Y*^W1^*Z*^W1^, *O*^W2^-*X*^W2^*Y*^W2^*Z*^W2^, the camera coordinate system *O*^C^-*X* ^C^*Y* ^C^*Z* ^C^ and the image coordinate system *O*^I^-*X*^I^*Y*^I^*Z*^I^ are defined on the two planar references, the camera and the image, respectively. Two checker-board-pattern references are considered as the transform bridges among the measured object, the laser plane and the camera.

The flexible laser plane intersects two planar references with two lines. According to the camera pinhole model^32^, the 3D points on the intersection lines and the projected image points are represented by

1 $${{\rm{A}}[{\rm{R}}}^{({\rm{W}},k)},{{\bf{t}}}^{({\rm{W}},k)}]{{\bf{X}}}_{i}^{({\rm{W}},k)}={s}^{(k)}{{\bf{x}}}_{i}^{({\rm{I}}{\rm{W}},k)}$$

where ${{\bf{X}}}_{i}^{({\rm{W}},{k})}={[{X}_{i}^{({\rm{W}},{k})},{Y}_{i}^{({\rm{W}},{k})},0,1]}^{{\rm{T}}}$ is the 3D point of the intersection laser line on the planar reference. ${{\bf{x}}}_{i}^{({\rm{IW}},{k})}={[{x}_{i}^{({\rm{IW}},{k})},{y}_{i}^{({\rm{IW}},{k})},1]}^{{\rm{T}}}$ is the projection point of ${{\bf{X}}}_{i}^{({\rm{W}},{k})}$ in the image coordinate system. ${{\rm{R}}}^{({\rm{W}},{k})}={[{r}_{pq}^{({\rm{W}},{k})}]}_{3\times 3}$, ${{\bf{t}}}^{({\rm{W}},{k})}={[{t}_{1}^{({\rm{W}},{k})},{t}_{2}^{({\rm{W}},{k})},{t}_{3}^{({\rm{W}},{k})}]}^{{\rm{T}}}$ are the rotation matrix and translation vector of the reference, from two world coordinate systems to the camera coordinate system. $${\rm{A}}=[\begin{array}{ccc}\alpha  & \gamma  & {u}_{0}\\ 0 & \beta  & {v}_{0}\\ 0 & 0 & 1\end{array}]$$ is the intrinsic parameters of the camera. ${{\rm{R}}}^{({\rm{W}},{k})},{{\bf{t}}}^{({\rm{W}},{k})}$ and A are generated from the Zhang’s method^33^. *s*^(*k*)^ is the scale factor. *k* = 1, 2 correspond to the coordinate systems *O*^w1^-*X* ^w1^*Y* ^w1^*Z* ^w1^, *O*^w2^-*X* ^w2^*Y* ^w2^*Z* ^w2^, respectively.

According to the projectivity in Eq. (1), the first and second coordinates of ${{\bf{X}}}_{i}^{({\rm{W}},{k})}$ are given by^33^

2 $$[\begin{array}{c}{X}_{i}^{({\rm{W}},{k})}\\ {Y}_{i}^{({\rm{W}},{k})}\\ 1\end{array}]={\{{\rm{A}}[{{\bf{r}}}_{p1}^{({\rm{W}},{k})}{{\bf{r}}}_{p2}^{({\rm{W}},{k})}{{\bf{t}}}^{({\rm{W}},{k})}]\}}^{-1}{s}^{(k)}{{\bf{x}}}_{i}^{({\rm{IW}},{k})}$$

where ${{\rm{R}}}^{({\rm{W}},{k})}=[{{\bf{r}}}_{p1}^{({\rm{W}},{k})}\quad {{\bf{r}}}_{p2}^{({\rm{W}},{k})}\quad {{\bf{r}}}_{p3}^{({\rm{W}},{k})}]$. Thus, ${{\bf{X}}}_{i}^{({\rm{W}},{k})}={[{X}_{i}^{({\rm{W}},{k})},{Y}_{i}^{({\rm{W}},{k})},0,1]}^{{\rm{T}}}$, and ${X}_{i}^{({\rm{W}},{k})},{Y}_{i}^{({\rm{W}},{k})}$ are derived from Eq. (2).

Therefore, the 3D point of the intersection laser line in the camera coordinate system is expressed by^34^

3 $${{\bf{X}}}_{i}^{({\rm{C}},k)}={[{\rm{R}}}^{({\rm{W}},k)},{{\bf{t}}}^{({\rm{W}},k)}]{{\bf{X}}}_{i}^{({\rm{W}},k)}$$

where ${{\bf{X}}}_{i}^{({\rm{C}},{k})}$ is the 3D point on the intersection laser line.

Let a point set ${{\bf{X}}}_{i}^{({\rm{C}})}=[{{\bf{X}}}_{i}^{({\rm{C}},1)},{{\bf{X}}}_{i}^{({\rm{C}},2)}]$. The points ${{\bf{X}}}_{i}^{({\rm{C}},1)}$ and ${{\bf{X}}}_{i}^{({\rm{C}},2)}$ are derived from different lines. Moreover, the 3D points ${{\bf{X}}}_{i}^{({\rm{C}})}$ of the intersection laser lines locate on the laser plane. Therefore,

4 $${({{\bf{X}}}_{i}^{({\rm{C}})})}^{{\rm{T}}}{\boldsymbol{\Pi }}={{\bf{0}}}_{2}$$

where **Π** = [*Π*_1_, *Π*_2_, *Π*_3_, 1]^T^ is the coordinate of the laser plane, which is determined by the point sets ${{\bf{X}}}_{i}^{({\rm{C}},1)}$ and ${{\bf{X}}}_{i}^{({\rm{C}},2)}$ on the two intersection laser lines. **0**_2_ is a 2-dimensional zero vector.

The set of the 3D points ${{\bf{X}}}_{i}^{({\rm{C}})}$ of the intersection laser lines is ${\rm{Q}}={[{{\bf{X}}}_{1}^{({\rm{C}})},{{\bf{X}}}_{2}^{({\rm{C}})},...{{\bf{X}}}_{i}^{({\rm{C}})},...,{{\bf{X}}}_{n}^{({\rm{C}})}]}^{T}$. As the points in Q are located on two different intersection lines, from Eq. (4), we have

5 $${\rm{Q}}{\boldsymbol{\Pi }}={{\bf{0}}}_{2n}$$

where **0**_2*n*_ is a 2*n*-dimensional zero vector. The laser plane **Π** can be solved by the singular value decomposition (SVD)^35^.

The 3D point ${{\bf{X}}}_{i}^{({\rm{M}})}$ on the measured object as well as on the laser plane obeys to^34^

6 $${({\boldsymbol{\Pi }})}^{{\rm{T}}}{{\bf{X}}}_{i}^{({\rm{M}})}=0$$

Moreover, in view of the camera pinhole model^32^, the 3D point ${{\bf{X}}}_{i}^{({\rm{M}})}$ on the measured object in the camera coordinate system also satisfies the projection

7 $${\rm{A}}{{\bf{X}}}_{i}^{({\rm{M}})}={s}^{({\rm{M}})}{{\bf{x}}}_{i}^{({\rm{IM}})}$$

where ${{\bf{x}}}_{i}^{({\rm{IM}})}={[{x}_{i}^{({\rm{IM}})},{y}_{i}^{({\rm{IM}})},1]}^{{\rm{T}}}$ are the 2D projection points of ${{\bf{X}}}_{i}^{({\rm{M}},{k})}$ in the image coordinate system. *s*^(M)^ is a scale factor.

**Figure 2:**
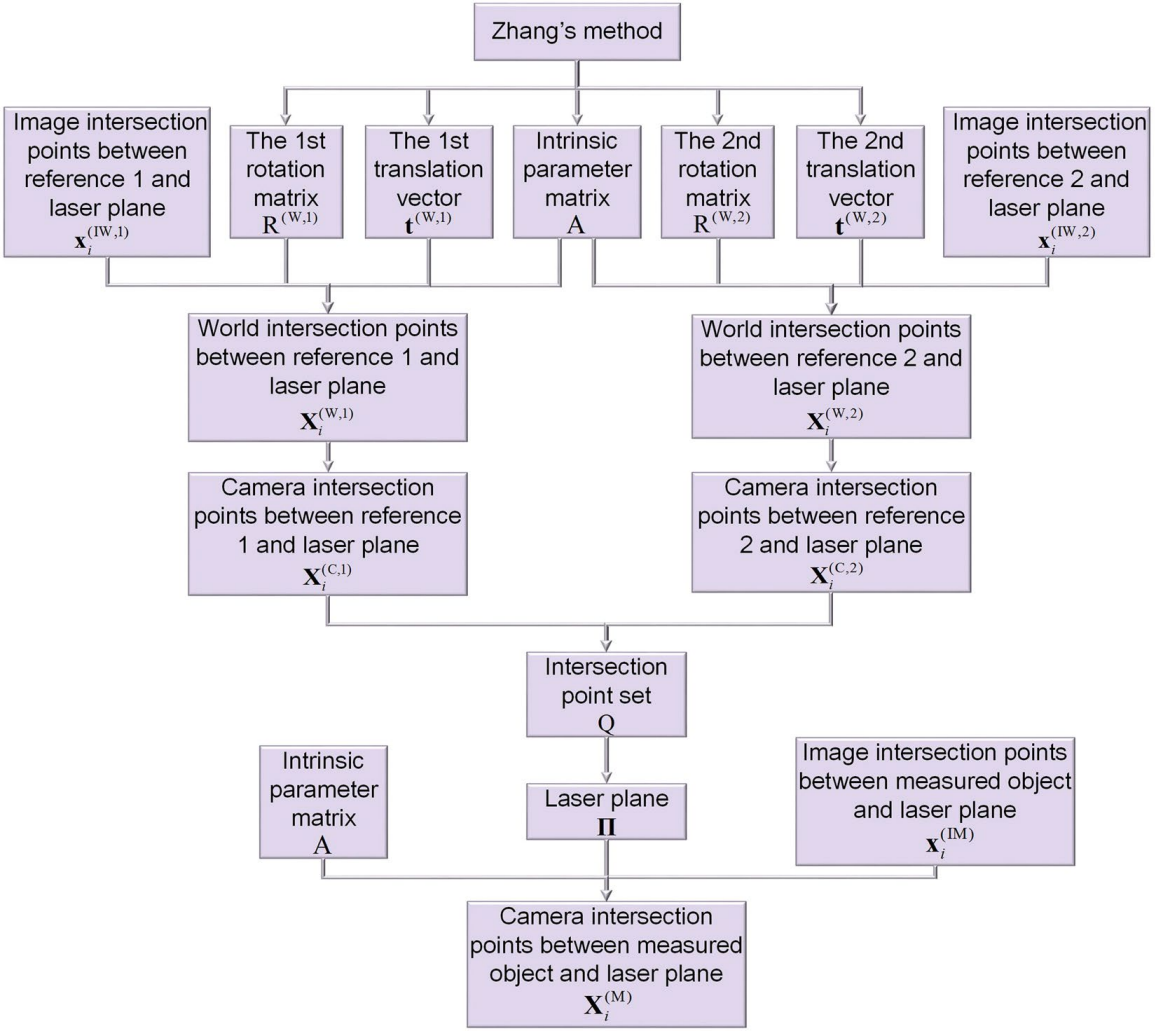
The closed form solution of the 3D reconstruction method adopting a flexible laser plane and two planar references.

The closed form solution of the 3D point ${{\bf{X}}}_{i}^{({\rm{M}})}$ on the measured object can be generated from stacking Eqs (6) and (7). The diagram of the closed form solution is shown in Fig. 2.

**Figure 3:**
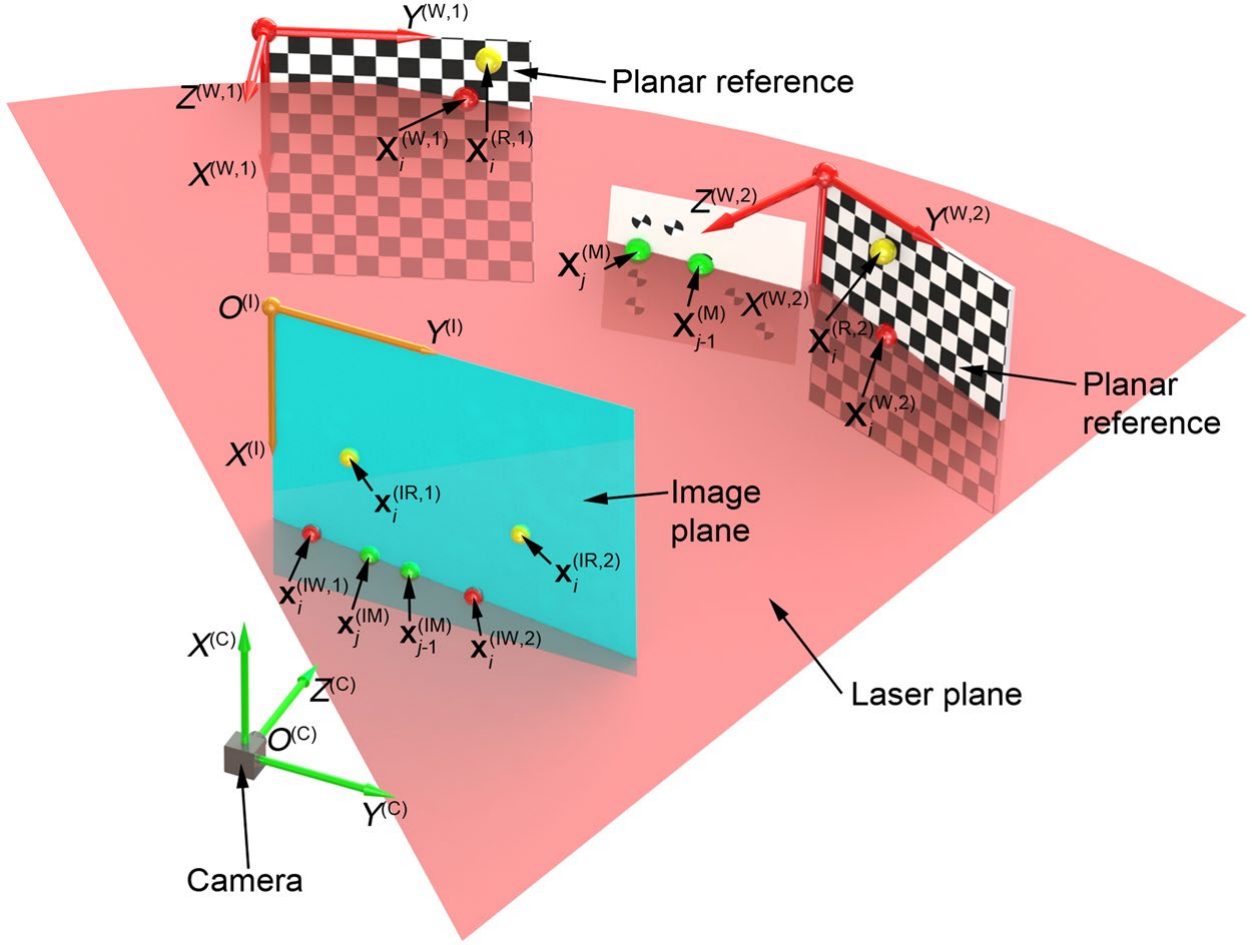
The optimization reconstruction adopting a target with the eight markers. Four distance benchmarks are generated from the eight markers.

In order to improve the reconstruction accuracy of the points on the measured object, the standard distance is employed as an optimization object to enhance the measurement accuracy^24^. In Fig. 3, we design a target for the benchmarks of the standard distances. There are eight different markers with the coordinate ${{\bf{X}}}_{j}^{({\rm{M}})}$ on the target. Four standard distances are given by the eight markers. The laser plane intersects two markers on the target. As the distance reconstructed by the laser plane should be equal to the real distance between two markers, the parameterized function is constructed by the difference between the distance on the target and the parameterized reconstruction distance given by

8 $$f(\alpha ,\beta ,\gamma ,{u}_{0},{v}_{0},{{\Pi }}_{1},{{\Pi }}_{2},{{\Pi }}_{3})=\sum _{j=1}^{m}[\parallel {{\bf{X}}}_{j}^{({\rm{M}})}(\alpha ,\beta ,\gamma ,{u}_{0},{v}_{0},{{\Pi }}_{1},{{\Pi }}_{2},{{\Pi }}_{3}){-{{\bf{X}}}_{j-1}^{({\rm{M}})}(\alpha ,\beta ,\gamma ,{u}_{0},{v}_{0},{{\Pi }}_{1},{{\Pi }}_{2},{{\Pi }}_{3})\parallel -{d}_{0}]}^{2}$$

where$$\begin{array}{rl}{X}_{j}^{({\rm{M}})}= & [\beta \alpha ({u}_{0}-{x}_{j}^{({\rm{I}}{\rm{M}})})-\gamma \alpha ({v}_{0}-{y}_{j}^{({\rm{I}}{\rm{M}})})]\{[\gamma \alpha ({v}_{0}-{y}_{j}^{({\rm{I}}{\rm{M}})})-\beta \alpha ({u}_{0}-{x}_{j}^{({\rm{I}}{\rm{M}})})]{{\Pi }}_{1}\\  & {-{\alpha }^{2}({v}_{0}-{y}_{j}^{({\rm{I}}{\rm{M}})}){{\Pi }}_{2}+{\alpha }^{2}\beta {{\Pi }}_{3}\}}^{-1}\\ {Y}_{j}^{({\rm{M}})}= & \alpha ({v}_{0}-{y}_{j}^{({\rm{I}}{\rm{M}})}){[({v}_{0}-{y}_{j}^{({\rm{I}}{\rm{M}})})(\gamma {{\Pi }}_{1}-\alpha {{\Pi }}_{2})-\beta {{\Pi }}_{1}({u}_{0}-{x}_{j}^{({\rm{I}}{\rm{M}})})+\alpha \beta {{\Pi }}_{3}]}^{-1}\\  & {Z}_{j}^{({\rm{M}})}=\alpha \beta {[\beta {{\Pi }}_{1}({u}_{0}-{x}_{j}^{({\rm{I}}{\rm{M}})})-\alpha \beta {{\Pi }}_{3}-({v}_{0}-{y}_{j}^{({\rm{I}}{\rm{M}})})(\gamma {{\Pi }}_{1}-\alpha {{\Pi }}_{2})]}^{-1}\end{array}$$ are the parameterized coordinates of the reconstruction point on the target.

**Figure 4:**
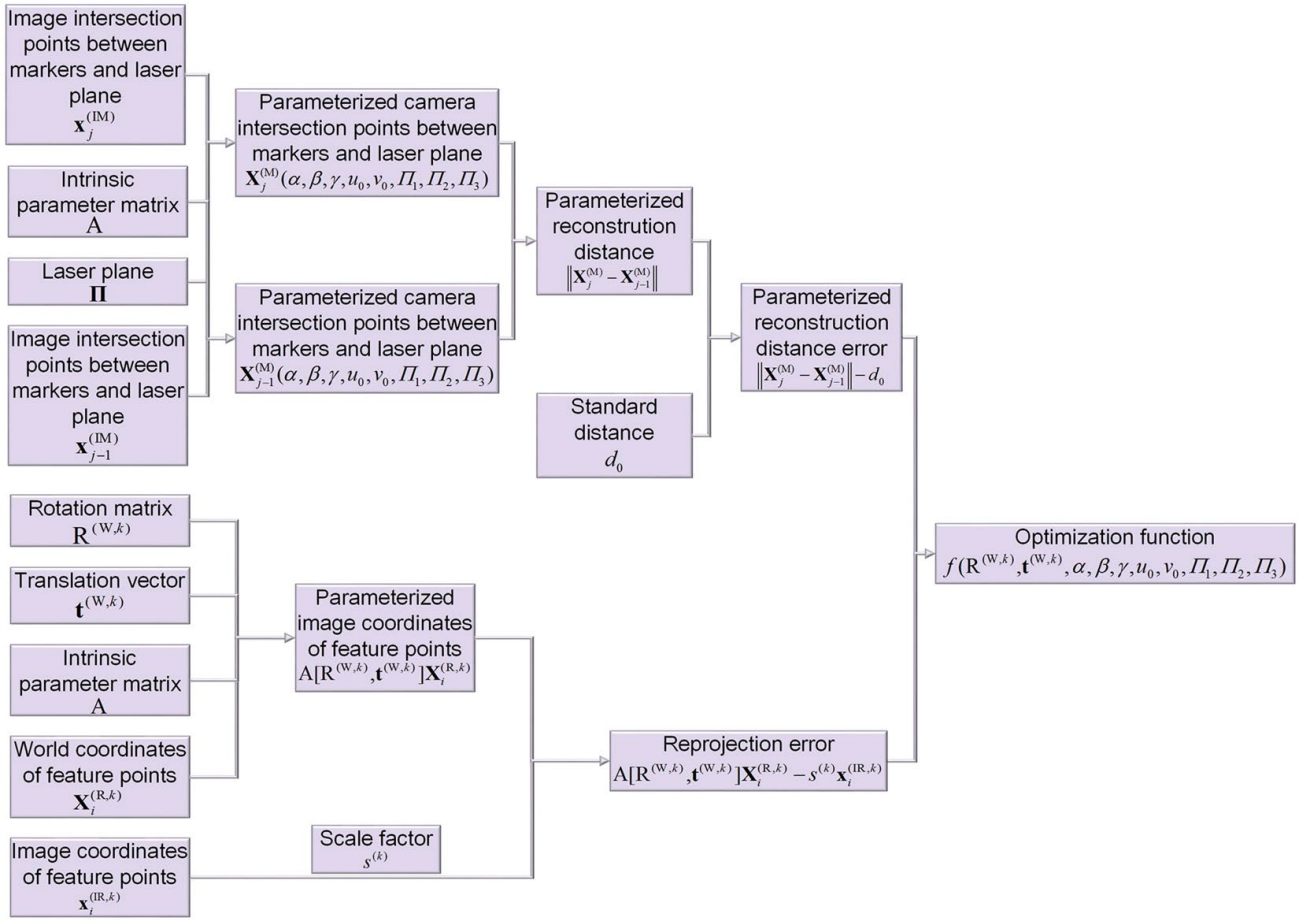
The optimization solution of the 3D reconstruction method adopting a flexible laser plane and two planar references.

Equation (8) covers the intrinsic parameters of the camera and the laser plane. However, the laser plane depends on the intersection laser points on the target, which is also determined by the extrinsic parameters R^(W,*k*)^ and **t**^(W,*k*)^ of the camera. Furthermore, the extrinsic parameters R^(W,*k*)^ and **t**^(W,*k*)^ represent the relative positions among the two planar references and the camera, as well as the coordinate of the laser plane. Therefore, on the basis of Eq. (8), we enhance the objective function by the parameterized reprojection errors with the extrinsic parameters. The final parameterized function is

9 $$\begin{array}{ccc}f({{\rm{R}}}^{({\rm{W}},k)},{{\bf{t}}}^{({\rm{W}},k)},\alpha ,\beta ,\gamma ,{u}_{0},{v}_{0},{{\Pi }}_{1},{{\Pi }}_{2},{{\Pi }}_{3}) & = & \sum _{j=1}^{m}\sum _{i=1}^{n}\{[\parallel {{\bf{X}}}_{j}^{({\rm{M}})}(\alpha ,\beta ,\gamma ,{u}_{0},{v}_{0},{{\Pi }}_{1},{{\Pi }}_{2},{{\Pi }}_{3})\\  &  & {-{{\bf{X}}}_{j-1}^{({\rm{M}})}(\alpha ,\beta ,\gamma ,{u}_{0},{v}_{0},{{\Pi }}_{1},{{\Pi }}_{2},{{\Pi }}_{3})\parallel -{d}_{0}]}^{2}\\  &  & +{\parallel {{\rm{A}}[{\rm{R}}}^{({\rm{W}},k)},{{\bf{t}}}^{({\rm{W}},k)}]{{\bf{X}}}_{i}^{({\rm{R}},k)}-{s}^{(k)}{{\bf{x}}}_{i}^{({\rm{I}}{\rm{R}},k)}\parallel }^{2}\}\end{array}$$

where ${{\bf{X}}}_{i}^{({\rm{R}},{k})}$, ${{\bf{x}}}_{i}^{({\rm{IR}},{k})}$ are the known 3D world coordinates and 2D image coordinates of the feature points on the two references. The optimized solutions of R^(W,*k*)^, **t**^(W,*k*)^, *α*, *β*, *γ*, *u*_0_, *v*_0_, *Π*_1_, *Π*_2_, *Π*_3_ are related to the minimized value of the parameterized function in Eq. (9). The optimized points on the measured object are solved by the optimized solutions. The diagram of the optimization solution is shown in Fig. 4.

## Results

Two 280 mm × 400 mm planar references are employed in the experiments. The references are covered with a check board pattern. The distance between the adjacent corner points on the planar reference is 20 mm. Each planar reference includes 247 feature points. The resolution of the captured images is 2048 × 1536 in the experiments. In the camera calibration, Harris corner recognition^36^ is adopted to acquire the coordinates of the feature points in the images of two planar references.

**Figure 5:**
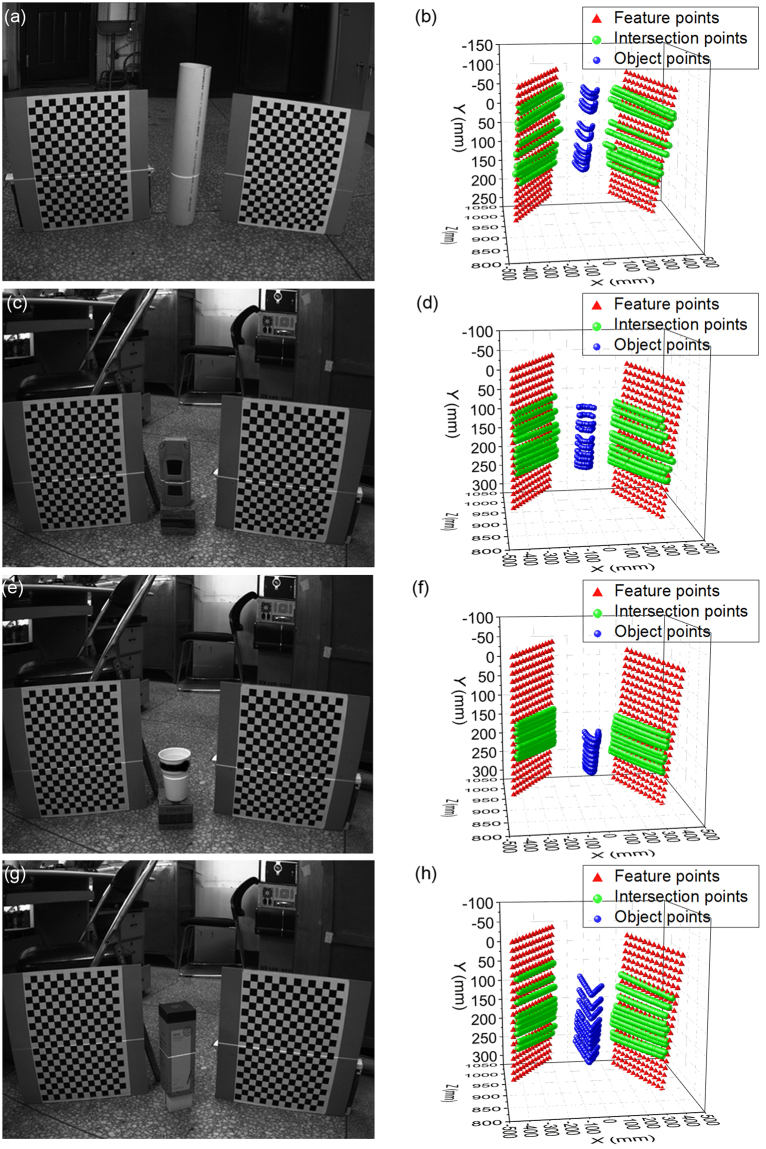
The reconstruction experiment results of the flexible laser plane and two planar references. (**a**) The reconstruction image of the cylindrical tube. (**b**) The point reconstruction results of the cylindrical tube. (**c**) The reconstruction image of the vehicle model. (**d**) The point reconstruction results of the vehicle. (**e**) The reconstruction image of the cup. (**f**) The point reconstruction results of the cup. (**g**) The reconstruction image of the box. (**h**) The point reconstruction results of the box.

The reconstruction experiment results of the flexible laser plane and two planar references are shown in Fig. 5. Figure 5(a,c,e and g) are the experimental photographs in the four groups of reconstruction experiments. A cylindrical tube, a vehicle model, a cup and a box are selected as the objects to be reconstructed. Figure 5(b,d,f and h) represent the point reconstruction results that are related to the Fig. 5(a,c,e and g). The red tetrahedrons show the distribution of feature points on the two planar references. The green spheres represent the points on the intersection lines between the flexible laser plane and the planar references in the camera coordinate system. The intersection line between the laser plane and the planar reference includes 40 green spheres. Two intersection lines determine the position of the laser plane. 10 different positions of the laser plane are selected in the four groups of the reconstruction experiments. The blue spheres illustrate the points on the intersection lines between the measured object and the flexible laser plane in the camera coordinate system. The blue curve is formed by 20 blue spheres on the intersection between the measured object and the laser plane.

In order to evaluate the precision of the reconstruction method, the eight different markers on the target in Fig. 3 are also considered as the benchmarks of the standard test distances. The relationships between the reconstruction errors and the standard test distances are analyzed by varying the standard distance. The standard test distance is determined by 20 mm, 30 mm, 40 mm and 50 mm, respectively. Four measurement distances between the camera and the object being measured are 900 mm, 1000 mm, 1100 mm and 1200 mm, respectively. The differences between the reconstructed distances and standard test distances are used to verify the accuracy of the profile reconstruction method. It is defined by^37^

10 $${E}_{k}=\Vert {{\bf{D}}}_{k}-{{\bf{D}}}_{k-1}\Vert -{D}_{0}$$

where *E*_*k*_ is the reconstruction error of the test distance, **D**_*k*_ and **D**_*k*−1_ are the reconstruction points of the markers, *D*_0_ is the standard test distance. 40 images of the target with the eight different markers are captured by the camera. 20 images of them are conducted to the optimization function. The other 20 images are chosen to evaluate the reconstruction error of the test distance.

**Figure 6:**
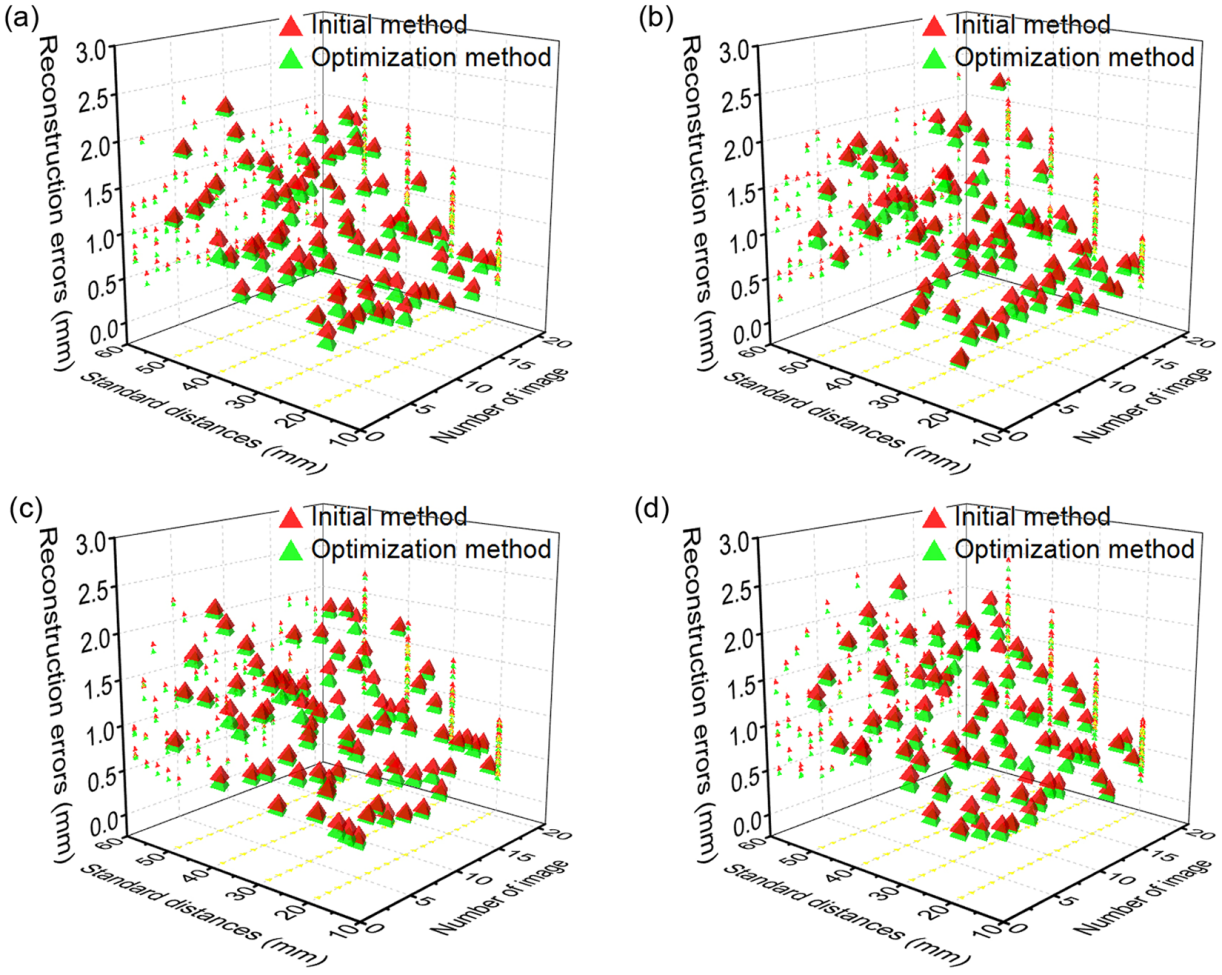
The experimental results of the reconstruction errors obtained from the initial method and the optimization method. The standard test distances are 20 mm, 30 mm, 40 mm and 50 mm, respectively. The measurement distances between the camera and the measured object are 900 mm, 1000 mm, 1100 mm and 1200 mm, which correspond to (**a**) to (**d**).

The reconstruction error results of the proposed optimization method are compared with ones of the initial closed-form-solution method in Fig. 6. The corresponding statistics are summarized in Table 1. The scopes of the reconstruction errors of the initial method are 0.21 mm–2.31 mm, 0.20–2.26 mm, 0.18 mm–2.22 mm and 0.17 mm–2.39 mm when the measurement distances between the camera and the measured object are 900 mm, 1000 mm, 1100 mm and 1200 mm. Moreover, the corresponding scopes of the optimization method are 0.16 mm–2.26 mm, 0.13 mm–2.21 mm, 0.13 mm–2.18 mm, 0.10 mm–2.28 mm. The average errors of the initial method are 1.02 mm, 0.98 mm, 1.00 mm and 1.04 mm when the measurement distances between the camera and the measured object are 900 mm, 1000 mm, 1100 mm and 1200 mm. The corresponding average errors of the optimization method are 0.94 mm, 0.90 mm, 0.92 mm and 0.95 mm. The root mean squares of errors of the initial method are 1.13 mm, 1.10 mm, 1.11 mm and 1.16 mm when the measurement distances between the camera and the measured object are 900 mm, 1000 mm, 1100 mm and 1200 mm. The corresponding root mean squares of the errors of the optimization method are 1.05 mm, 1.03 mm, 1.04 mm and 1.08 mm.

**Table 1:** The reconstruction errors of the initial method and the optimization method.

Measurement distance	Standard test distance	Solution method	Error			
			Mean	RMS	Min.	Max.
900 mm	20 mm	Initial method	0.52	0.54	0.21	0.78
		Optimization method	0.44	0.46	0.16	0.65
	30 mm	Initial method	0.86	0.90	0.42	1.39
		Optimization method	0.79	0.83	0.35	1.32
	40 mm	Initial method	1.19	1.24	0.57	1.78
		Optimization method	1.08	1.14	0.47	1.70
	50 mm	Initial method	1.52	1.57	0.70	2.31
		Optimization method	1.46	1.50	0.66	2.26
1000 mm	20 mm	Initial method	0.48	0.50	0.20	0.70
		Optimization method	0.41	0.43	0.13	0.65
	30 mm	Initial method	0.83	0.87	0.41	1.30
		Optimization method	0.76	0.81	0.31	1.28
	40 mm	Initial method	1.12	1.19	0.42	1.75
		Optimization method	1.02	1.09	0.36	1.67
	50 mm	Initial method	1.49	1.55	0.78	2.26
		Optimization method	1.41	1.48	0.70	2.21
1100 mm	20 mm	Initial method	0.49	0.53	0.18	0.81
		Optimization method	0.43	0.47	0.13	0.78
	30 mm	Initial method	0.84	0.88	0.42	1.39
		Optimization method	0.77	0.82	0.34	1.33
	40 mm	Initial method	1.16	1.21	0.52	1.73
		Optimization method	1.06	1.11	0.47	1.67
	50 mm	Initial method	1.51	1.56	0.83	2.22
		Optimization method	1.44	1.49	0.77	2.18
1200 mm	20 mm	Initial method	0.53	0.56	0.17	0.82
		Optimization method	0.45	0.48	0.10	0.71
	30 mm	Initial method	0.88	0.92	0.33	1.40
		Optimization method	0.81	0.85	0.31	1.33
	40 mm	Initial method	1.22	1.27	0.51	1.88
		Optimization method	1.09	1.16	0.29	1.72
	50 mm	Initial method	1.55	1.62	0.64	2.39
		Optimization method	1.47	1.53	0.59	2.28

In the test with the standard distance of 20 mm, the ranges of the minimum value, the maximum value, the mean and the root mean square of the reconstruction errors of the initial method are 0.17 mm–0.21 mm, 0.70 mm–0.82 mm, 0.48 mm–0.53 mm and 0.50 mm–0.56 mm. However, the ones of the optimization method are 0.10 mm–0.16 mm, 0.65 mm–0.78 mm, 0.41 mm–0.45 mm and 0.43 mm–0.48 mm. In the test with the standard distance of 30 mm, the scopes of the minimum value, the maximum value, the mean and the root mean square of the reconstruction errors of the initial method are 0.33 mm–0.42 mm, 1.30 mm–1.40 mm, 0.83 mm–0.88 mm and 0.87 mm–0.92 mm. Then, the ones of the optimization method are 0.31 mm–0.35 mm, 1.28 mm–1.33 mm, 0.76 mm–0.81 mm and 0.81 mm–0.85 mm. In the test with the standard distance of 40 mm, the ranges of the minimum value, the maximum value, the mean and the root mean square of the reconstruction errors of the initial method are 0.42 mm–0.57 mm, 1.73 mm–1.88 mm, 1.12 mm–1.22 mm and 1.19 mm–1.27 mm. However, the ones of the optimization method are 0.29 mm–0.47 mm, 1.67 mm–1.72 mm, 1.02 mm–1.09 mm and 1.09 mm–1.16 mm. The scopes of the minimum value, the maximum value, the mean and the root mean square of the reconstruction errors of the initial method are 0.64 mm–0.83 mm, 2.22 mm–2.39 mm, 1.49 mm–1.55 mm and 1.55 mm–1.62 mm with the standard distance of 50 mm. Moreover, the ones of the optimization method are 0.59 mm–0.77 mm, 2.18 mm–2.28 mm, 1.41 mm–1.47 mm and 1.48 mm–1.53 mm.

The relative errors of the initial method are 2.59%, 2.88%, 2.96% and 3.04% while the relative errors of the optimization method are 2.19%, 2.62%, 2.69% and 2.91%, when the measurement distance between the camera and the measured object is 900 mm and the standard test distances are 20 mm, 30 mm, 40 mm and 50 mm. When the measurement distance between the camera and the measured object are 1000 mm, the relative errors of the initial method are 2.40%, 2.78%, 2.79% and 2.99% while the relative errors of the optimization method are 2.03%, 2.54%, 2.55% and 2.83%, in the test with the standard distances of 20 mm, 30 mm, 40 mm and 50 mm. The relative errors of the initial method are 2.47%, 2.81%, 2.91% and 3.02% while the relative errors of the optimization method are 2.13%, 2.58%, 2.64% and 2.88% when the measurement distance between the camera and the measured object is 1100 mm. The relative errors of the initial method are 2.65%, 2.92%, 3.04% and 3.11% while the relative errors of the optimization method are 2.25%, 2.69%, 2.71% and 2.94%, with the measurement distance of 1200 mm and the standard test distances of 20 mm, 30 mm, 40 mm and 50 mm.

**Figure 7:**
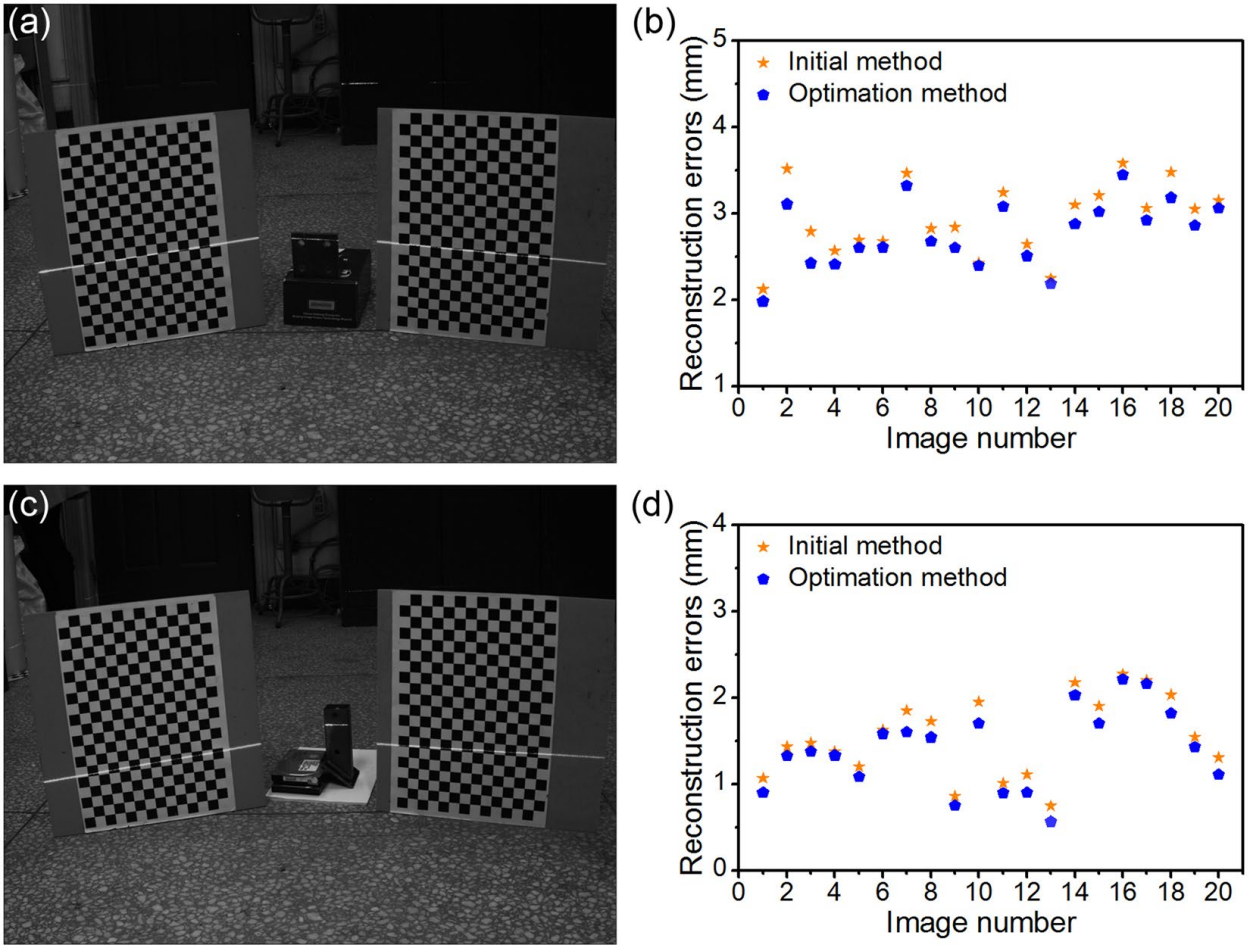
The reconstruction examples of two mechanical parts with the flexible laser plane and two planar references. (**a**) The reconstruction example of the first mechanical part. (**b**) The point reconstruction errors of the first example. (**c**) The reconstruction example of the second mechanical part. (**d**) The point reconstruction errors of the second example.

Two examples in Fig. 7 are provided for the measurement of mechanical parts by using the described method. The true values of the two mechanical parts, which are measured by a vernier caliper, are 90.16 mm and 49.50 mm, respectively. Nevertheless, the mean errors of the initial method and the optimization method obtained from the different 20 images of the first measured part are 2.94 mm and 2.77 mm. The average relative errors of the initial method and the optimization method are 3.26% and 3.07%. The average errors of the initial method and the optimization method of the second measured mechanical part are 1.55 mm and 1.40 mm. The average relative errors of the initial method and the optimization method are 3.12% and 2.84%, respectively.

## Discussion

In this paper, a 3D reconstruction process is realized by the flexible laser plane without position limitation relative to the camera and the bi-planar references. In order to calibrate the flexible laser plane relative to the camera, 3D and 2D targets are naturally considered as the references to solve the flexible laser plane. The 3D target could be designed with the combination of two orthogonal planes or the two planes with the known relative position. This kind of target is difficult to be manufactured. And then, we solve the flexible laser plane by the 2D target. However, a flexible laser plane cannot be reconstructed from only one planar target. It is because there is only one intersection line between the laser plane and the planar target, and a laser plane cannot be determined by only one line. In view of the above reasons, two planar targets are chosen as the references to reconstruct the flexible laser plane. As there are two intersection lines between the laser plane and the two planar targets, the flexible laser plane is derived from the two intersection lines. In this method, the laser plane is flexible to the camera and there is no strict restriction of the position between two planar targets.

From the experimental results, we naturally come to the conclusion that the reconstruction errors of the optimization method are generally smaller than those of the initial method. It is to say, the optimization method is closer to the real 3D value than the initial method. This proves that the optimization function effectively reduces the reconstruction experimental error and contributes good stability and reliability. In addition, the relative errors of the optimization method are smaller than those of the initial method. Furthermore, the relative errors of the two methods are less than 4%. The averages, the root mean squares and relative errors present steady growing trends with the increasing standard test distance from 20 mm to 50 mm. As the measurement distance between the camera and the measured object rises from 900 mm to 1200 mm, the distance reconstruction errors decrease firstly, then increase gradually. It is worth noting that the averages and the root mean squares of the distance reconstruction errors achieve the smallest values when the measurement distance between the camera and the measured object is 1000 mm. The averages and the root mean squares of the distance reconstruction errors are the largest values when the camera is 1200 mm away from the object being measured in the experiments. The averages and the root mean squares of the distance reconstruction errors under the measurement distance of 1100 mm are slightly smaller than those under the distance of 900 mm. Therefore, the experimental results show that the reconstruction results are the closest to the true values when the camera is 1000 mm away from the object being measured and the standard test distance is 20 mm. Two mechanical parts are measured by the method. The average relative errors of the initial method and the optimization method are less than 5%. As the laser projector is small and flexible to the camera, the measurement just requires the laser projector to approach the measured object. Therefore, the measurement can be achieved in a small space by the laser projector. The method has potential applications in the object profile measurement fields, such as automotive industry and aviation industry.

### Summary

An optimization reconstruction method of object profile is realized in this paper by using a flexible laser plane and bi-planar references. Two planar references are adopted to build the transformations among the measured object, the laser plane and the camera. Two world coordinate systems are determined by the two planar references. Therefore, the camera internal parameters, the rotation matrix and the translation vector are determined by the projections from the feature points on the two planar references to ones on the image. The laser plane is modeled by the camera coordinates of the points on the intersection lines between the laser plane and two planar references. The camera coordinates of the points on the intersection curves of the measured object are obtained by the laser plane and the projection relationship of the camera. A target taking the markers with different distances is adopted to evaluate the reconstruction accuracy of the 3D points on the measured object. Then, an optimization function is established to enhance the accuracy of the reconstruction results by minimizing the parameterized differences of the standard test distances and the reconstruction distances and minimizing the parameterized reprojection errors of the feature points on two planar references. The effects of the measurement distance between the camera and the measured object, and the ones of the standard test distance on the target are investigated by the experiments. The mean of the reconstruction error of the initial method is 1.01 mm while the mean of the reconstruction error of the optimization method is 0.93 mm. The root mean square of the reconstruction errors of the initial method is 1.12 mm while the root mean square of the reconstruction errors of the optimization method is 1.05 mm. Furthermore, the means of the relative errors of the initial method and the optimization method are 2.84% and 2.57%, respectively. Therefore, the optimization method effectively improves the accuracy of the reconstruction method and provides the relative errors smaller than 4%. Two mechanical parts are measured by the method to explain the applications. The reconstructed values are compared with the true values from a vernier caliper. The average relative errors of the initial method and the optimization method are less than 5%. Consequently, the proposed optimization reconstruction method, using the bi-planar references and a flexible laser plane, has potential applications in the object profile measurement fields.

### Data availability

The datasets generated during the current study are available from the corresponding author on reasonable request.
